# Progress on Thin Film Freezing Technology for Dry Powder Inhalation Formulations

**DOI:** 10.3390/pharmaceutics14122632

**Published:** 2022-11-28

**Authors:** Sagar R. Pardeshi, Eknath B. Kole, Harshad S. Kapare, Sachin M. Chandankar, Prashant J. Shinde, Ganesh S. Boisa, Sanjana S. Salgaonkar, Prabhanjan S. Giram, Mahesh P. More, Praveen Kolimi, Dinesh Nyavanandi, Sathish Dyawanapelly, Vijayabhaskarreddy Junnuthula

**Affiliations:** 1 Department of Pharmaceutics, St. John Institute of Pharmacy and Research, Palghar 401404, India; 2 University Institute of Chemical Technology, KBC North Maharashtra University, Jalgaon 425001, India; 3 Department of Pharmaceutics, Dr. D. Y. Patil Institute of Pharmaceutical Sciences and Research, Pune 411018, India; 4 Department of Pharmaceutics, R. C. Patel Institute of Pharmaceutical Education and Research, Shirpur 425405, India; 5 Department of Pharmaceutical Sciences, University at Buffalo, The State University of New York, Buffalo, NY 14214, USA; 6 Department of Pharmaceutics, Dr. Rajendra Gode College of Pharmacy, Buldhana 443101, India; 7 Department of Pharmaceutics and Drug Delivery, University of Mississippi, Oxford, MS 38677, USA; 8 Product Development, Continuus Pharmaceuticals, 25 Olympia Ave, Woburn, MA 01801, USA; 9 Department of Pharmaceutical Sciences and Technology, Institute of Chemical Technology, NP Marg, Matunga, Mumbai 400019, India; 10 Drug Research Program, Faculty of Pharmacy, University of Helsinki, Viikinkaari 5 E, 00790 Helsinki, Finland

**Keywords:** thin film freezing, dry fine powder, novel drug delivery, poorly soluble drug, pulmonary, inhalation

## Abstract

The surface drying process is an important technology in the pharmaceutical, biomedical, and food industries. The final stage of formulation development (i.e., the drying process) faces several challenges, and overall mastering depends on the end step. The advent of new emerging technologies paved the way for commercialization. Thin film freezing (TFF) is a new emerging freeze-drying technique available for various treatment modalities in drug delivery. TFF has now been used for the commercialization of pharmaceuticals, food, and biopharmaceutical products. The present review highlights the fundamentals of TFF along with modulated techniques used for drying pharmaceuticals and biopharmaceuticals. Furthermore, we have covered various therapeutic applications of TFF technology in the development of nanoformulations, dry powder for inhalations and vaccines. TFF holds promise in delivering therapeutics for lung diseases such as fungal infection, bacterial infection, lung dysfunction, and pneumonia.

## 1. Introduction

Recently, the dissolution profile of water-insoluble medications has been significantly improved by using the particle engineering technique known as thin film freezing (TFF) [1]. TFF is the evolution of a fast-freezing technique to form films and produce powdered drug particles. The API and stabilizer solution are immediately iced onto a cryogenically frozen surface in the TFF process, after which the frozen particles are collected, and the solvent is sublimated. The supercooling of the API and stabilizer solution minimizes the phase separation and nucleation, which possibly converts the crystalline drug to an amorphized form [2]. Additionally, the high freezing rate increases the number of liquid crystals and lowers the particle size. The amorphous composition with enhanced surface area contributes to an increased rate of drug dissolution. Zhang et al. formulated a fenofibrate (FB) solid dispersion through TFF to enhance drug solubility and bioavailability. The dissolution study showed more than 80% fenofibrate release within 30 min. The in vivo animal study showed the shortest T_max_ of 1.42 h, which is similar to the in vitro data obtained for FB solid dispersion [3].

Pharmaceutical formulations such as liquids or powders are used to deliver medications designed for inhalation into the lungs. Most of them have been produced as liquids, whereas powder formulations have various advantages, especially for proteins and other biological products [1]. Pharmaceutical dry powders typically have better aerosol characteristics, circumvent the cold chain, are more stable, have reduced dosage needs, and are easier to administer. The production of biologics as powders is especially advantageous for costlier biologics because the dry powder state often increases the shelf life of biologics and lowers the expense of their transport and storage. Powder preparation methods include spray-drying (SD), traditional shelf freeze-drying (shelf FD), spray freeze-drying (SFD), and spray freezing into liquid (SFL). However, all approaches in the context of biologics result in some extent of the biologics’ agglomeration, denaturation, and/or loss of functionality. Additionally, these techniques result in powder particles with limited surface area, low efficiency, and/or a wide size distribution, which are not suitable for pulmonary delivery by inhalation, especially of biologics [4].

TFF technology has been applied to improve the physical characteristics of drug particles. The solid powdered flow characteristics were improved and widely used for the development of a dry powder inhaler (DPI). The aerodynamics of the powdered material were promising in comparison to other processing steps. The particle shape, size, density, and porosity were modified after being processed using TFF. The TFF process generally provides low-density particles with perforated matrices that are easy for solvent interactions and aerosol properties. The brittle nature of particles can easily be transported across the spray nozzle of aerosols, providing outstanding behavior. Quick supercooling of drug-carrier liquids converted to stable mass with sublimed solvent vapors. This further follows lyophilization to eliminate the solvents used during the process. Aqueous or solvent mixtures with higher freezing points are considered suitable for TFF [5]. TFF methodology has recently been used by Hufnagel and colleagues [6] to create dry monoclonal antibody powders that can be aerosolized. In this study, a viable dry powder composed of an anti-programmed cell death protein (anti-PD-1 mAb) was prepared by processing with the TFF technique. The supportive base containing lactose/leucine (60:40 *w*/*w*) acted as a carrier dissolved in phosphate-buffered saline for homogenization. The dried particles were in the form of nanoaggregations with high porosity. The potential to elevate the *Tg* to 152 °C with the inclusion of polyvinylpyrrolidone (PVP) K40 has indeed improved the mAb storage stability. The study suggests that TFF is a very promising technique for the processing of biomolecules without disturbing its potency and produces stable DPI for pulmonary applications.

Considering the widespread potential of TFF technology in drug delivery, the present review highlights the fundamental aspects of TFF for the development of dry powdered material for pulmonary delivery. Understanding the physicochemical properties of powdered material prepared by TFF used to enhance solubility, dissolution, and aerosolization properties of API. The TFF technique is not only suitable for pulmonary delivery but also suitable for parental administration as a reconstitution.

## 2. Fundamentals of Thin Film Freezing Technology

**Figure 1:**
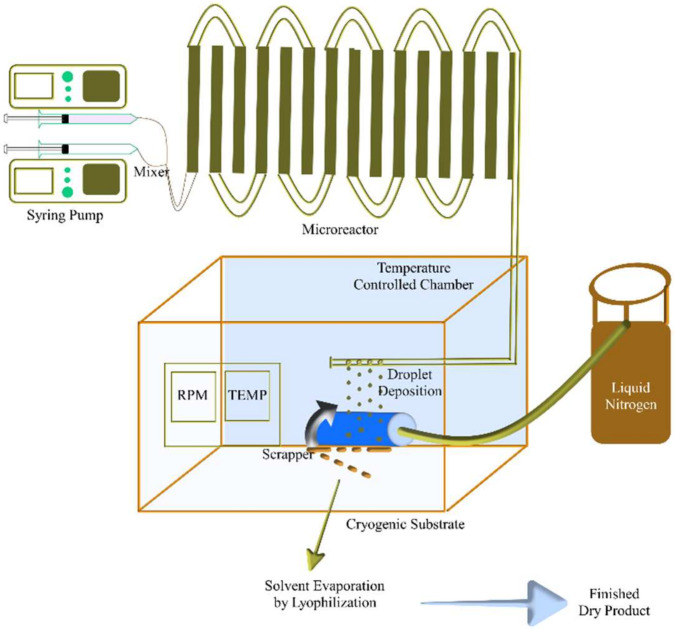
Model setup of the TFF system.

Most of the drugs available have issues of poor water solubility that cannot be administered via the oral route. In the case of biological products and vaccines, the challenges include storage and handling at a lowered temperature [7,8]. TFF technology converts drugs into a more solubilized and stable powder form, which also addresses the major challenges mentioned above [4]. Thin film freezing to produce pharmaceutical powders is an evolution of earlier freezing processes on cold surfaces to form films. The electronics and semiconductor sectors often use deposition and crystal growth of fluid droplets onto a chilled solid object to apply tiny coatings of frozen substance to a substrate. The TFF involves the transfer of solution containing API and stabilizer processes from any processing equipment, such as a microchannel reactor (used for continuous manufacturing representation), and deposited on the cryogenically cooled solid surface supplied with liquid nitrogen. Continuous rolling provides a larger surface area for the freezing of liquid, as represented in Figure 1. Recent research indicates that dropping different solutions from approximately 10 cm into a spinning steel drum is sufficient for the freezing process. The TFF equipment is kept in a controlled environment chamber specifically in a dry box with a humidity level of less than 10% to reduce the production of vapor condensation on the steel substrate. The revolving drum transforms into a continual freezing process, giving it scalability benefits over other freezing-induced particulate methods, although maintaining a low humidity raises operational expenses. The amounts of cryogen needed to make mass production batch sizes can also raise the price of the product with all freezing techniques. Exactly which of the freezing procedures is the most cryogenically effective is unknown. As the liquid droplet impinges on the cryogenic surface, a thin disc or wafer is formed and rapidly cooled until frozen. Before making a full rotation, the iced disc is brushed off the top with a blade (scrapper) and dropped into a receiving pan. The received dried substrate was chilled with dry ice or liquid nitrogen. After a batch has been processed, the pan with the frozen plates is moved to a tray lyophilizer, in which the solvent is evaporated [2,9,10,11]. The rapid freezing technologies previously reported have solvent selection based on the solubility of the solute and the ease of lyophilization. The fluid mechanics of the liquid after spreading and freezing must be considered to affect the characteristics of the powder. The selection of solvent is critical in TFF and governs many challenges. The speed at which the thin disc cooling depends on how moist the substrate is or how easily an impeding droplet can form a narrow disc with a large circumference before freezing [2]. Furthermore, feed systems for faster heat transfer are more desired when using solvents with higher thermal diffusion coefficients. These limitations may prevent certain compounds from benefiting from the process since an ideal solvent with specialized properties may not exist or may be toxic to the environment or patient at residual/trace levels. Unpublished research by Overhoff has been performed to circumvent these limits by creating cosolvent systems that reduce the solubility limitations of a particular solvent while maintaining a few of the ideal fluid dynamic properties. Itraconazole TFF particles have been produced using a different combination of 1,3-dioxolane (a solubility enhancer) in tertbutanol (a solvent with optimal freezing and spreading characteristics), whereas a feed solution containing tacrolimus has improved qualities using acetonitrile in water [12]. Combinations of methylene chloride, p-xylene, and 1,4-dioxane have been used with similar capacities [1]. The advantages and limitations of TFF over other techniques to produce DPI are described in detail in Table 1.

In pharmaceutical formulation development, the powder dosage forms are mostly preferred over liquid due to good stability, low dose requirements, convenience in administration, and avoidance of the cold chain. Protein delivery is complicated and expensive. Powders were also found to possess good aerosol properties in the case of pulmonary delivery. The conventional techniques of powder formulations, such as spray-drying and freeze-drying, give powder with poor aerosol properties. Powders with TFF technology are found to be better in terms of morphology, solubility, stability, dissolution profile, and aerosol properties. In the case of biological materials, this method also permits efficient pulmonary administration and cold chain-free preservation [4].

## 3. Characteristics of Nanoparticles Produced by TFF

Many medications are poorly soluble and cannot be properly administered by oral as well as other routes. As a result, numerous strategies have been created to address the problems related to low solubility and bioavailability. It included surface solid dispersion, spray drying, cospray drying, melt mixing, TFF, evaporative precipitation into aqueous systems, freeze drying, etc. [14]. These issues arise from the other types of dosage forms, such as vaccines, sera, toxoids, etc., being administered parenterally and can provide logistical issues due to the need for cold storage and proper handling [15]. All these problems are resolved by TFF, which creates medications that are incredibly soluble and stable in dry powder. There is a great need for innovative solutions that boost the stability of biological drugs and vaccines and enhance the solubility and dissolution characteristics. Additionally, advanced techniques convert the liquid formulation to a solid powder and reduce the load on the cold chain of transportation and handling [16]. Moreover, TFF typically yields high-potency powders with few inactive components, which is advantageous for medication administration. When the drug solution encounters the cryogenic surface, it flashes and freezes, producing “drug ice” with an amorphous nature. Lyophilization removes the solvent system, resulting in highly porous “brittle matrix particles” with large surface areas and low bulk densities. Particle morphology can be changed during the TFF process and requires understanding the fluid dynamics of liquid droplets depositing on the surface with simultaneous heat transfer characteristics from the roller surface. The fluid dynamics study provides spreadability characteristics of liquid droplets and interactions between cold surfaces and liquids. When brittle matrix particles encounter the target site, supersaturation occurs as sublimation of the solvent is initiated. The aerodynamic properties of TFF particles contained in dry powder inhalers (DPIs) enable up to 80% of the medication to be distributed in the deep lung. For a range of various routes of administration, such as intranasal, intraocular, and transdermal, larger therapeutic benefits can be obtained at substantially lower doses since the API is trapped in an extremely active state [17]. Biologics and vaccines that have undergone TFF conversion can be transported at room temperature and reconstituted at the site of administration, doing away with the need for a cold chain. TFF technology is useful in the creation of biologics and vaccines, as well as medications for indications other than lung disorders, in animal models, and in vitro testing [18]. An in vivo absorption study evaluating the TFF-treated powder with brand-name Sporanox^®^ was then carried out. The ultrarapid freezing (URF) technique is used to produce high surface area particles made from solid solutions. The physicochemical properties of APIs are reported to be improved by rapid freezing techniques, enhancing bioavailability [2]. However, it is still unclear what effect various freezing patterns and rates will have on the URF procedure. As a result, this research examined how the characteristics of micronized Danazol powders are affected by the solvent property and droplet-thin film shape. The tacrolimus-containing solid dispersion (TSD) was made into amorphous nanoparticles using the TFF method. The study sought to determine the effect of the stabilizer combination on the supersaturation of tacrolimus. The combination of various sugar stabilizers, such as lactose, mannitol, and trehalose, at variable concentration levels was considered to understand the supersaturation effect of TSDs [19]. Batch F6 demonstrated a smaller MMAD and a greater FPF than F8 and F9 at 95% drug loading (*p* < 0.05), demonstrating that lactose performs better in aerosols than mannitol and trehalose.

### 3.1. Solubility and Dissolution

The powdered characteristics of the material were brittle after being processed using TFF, with additional findings suggesting the homogenous distribution of drug within the carrier matrix. The process converts crystalline drugs to an amorphous state in the presence of polymeric interactions. The dissolution test of TAC treated with and without lactose (5%) suggests variability in the release rate. The treated TAC releases 85% of the drug at the end of 6 h, while untreated TAC only releases 30% of the drug [19]. The variable ratios of TAC and lactose at 50:50 and 95:5 show similar performance if processed using the TFF technique. The dissolution characteristics of the TFF-processed TAC formulation show a similar release pattern [20]. The rate of dissolution was also increased after combining crystalline drugs with the amorphous formulation. The amorphous solid dispersion can alter the amorphized state to a crystalline form at elevated temperature and humidity conditions, which substantially affects the in vivo performance of crystalline drugs and reduces bioavailability. The crystalline drug has much less internal pressure for interaction with solvent molecules, leading to lower dissolution and solubility. The amorphous product increased the saturation levels and improved the biophysical properties of the crystalline drug. A contemporary study may help to understand the storage conditions and effects on the crystallization behavior of tacrolimus-like drugs [21]. After TFF processing, TAC shows a high degree of solubility and rate of dissolution. The solubility characteristics of amorphous TAC after processing through the TFF technique show a 35-fold increase. Another study examined how well-tolerated TFF remdesivir formulations with various excipients (Captisol^®^, mannitol, lactose, and leucine) dissolved in comparison to untreated and plain TFF remdesivir [14]. TFF TAC formulations dissolved more easily due to enhanced amorphous TAC solubility. Drugs possess higher solubility and dissolution rates and dissolve faster in their amorphous form because they have a greater free energy state [21,22]. In some cases, adding a polymer to a TFF formulation as an excipient might improve and extend supersaturated solubility in addition to speeding up and increasing the rate of dissolution. The degree of supersaturation (C/Ceq) was calculated by considering the ratio of the concentration of the drug dissolved to the equilibrium solubility of the drug (Ceq) of crystallites [22]. According to Yang et al., itraconazole dispersion containing mannitol: lecithin (0.5:2) could improve the degree of supersaturation. The colloidal dispersion containing itraconazole shows a minimum of up to 22 times and a maximum of 27 times equilibrium solubility achieved for 5 and 15 min, respectively [23,24]. The physical mixture containing itraconazole in the presence of mannitol and lecithin only reached up to 22 times at the end of 15 min. The degree of supersaturation was decreased after 2 h and was comparatively much less stable than itraconazole prepared using TFF [23]. In a two-stage dissolution test under acidic pH, Overhoff and associates performed a comparative evaluation of TFF tacrolimus-sodium dodecyl sulfate formulation and commercial tacrolimus capsules (Prograf^®^). The TFF containing tacrolimus shows five-fold higher supersaturation in comparison to Prograf^®^. The increased supersaturation level of tacrolimus was due to the presence of sodium dodecyl sulfate, and TFF processing led to an increase in supersaturation from 17- to 22-fold at pH 1.2 and 6.8. The TFF-containing tacrolimus did not precipitate. Comparatively, Prograf^®^ precipitated after the change in pH, which may be due to a decrease in the degree of supersaturation [9]. Proteins prepared as powders instead of liquids have the benefit of being less restricted by the protein’s solubility in the dose. Excipients such as cyclodextrin are considered excellent candidates for the delivery of proteins. The excipients increase solubility via adsorption at the surface and reduce agglomeration at the air–liquid interface [25]. For the accumulation of proteins at the air–liquid interface area, surfactants, ethanol, acidic conditions, etc., are typically being verified. To improve solubility and stability, certain additives are being used. Additives also help to control the release and dissolution time of proteins. Poly(D,L-lactide glycolcolide) (PLG) or PLG/poloxamer microspheres can effectively be used for the delivery of proteins. Cryoprotectants such as trehalose may be used during the drying process to avoid loss of proteins [26]. The PLG-conjugated BSA released 80% of the protein within 4 h, while the addition of poloxamer extended the release of proteins by another 1–2 days. The poloxamer-to-PLG ratio plays a major role in extending the release of protein. The application of TFF for the preparation of dry powder not only increases the flow characteristics of vaccines but also promotes dissolution inside lung tissue.

### 3.2. Stability of TFF Powders

The ultra-rapid freezing technique nurtures the aerodynamic properties of the powdered material. The aerosol efficiency was improved by 95%. The dissolution characteristics were also shown to be improved, with a slight effect observed in the water sorption capacity. Additionally, TFF processing varies the specific surface area, which may be due to the instant freezing of the solution. The stability of various pharmaceutical TFF powders has been analyzed in terms of several studies, and it is well mentioned in several literary studies that many crystalline natures of compounds are highly stable compared to their amorphous natural counterparts. In summary, the TFF approach was used to create crystalline VCZ/MAN (95:5 *w*/*v*) nanoaggregates that have been physiochemically robust after being stored at ambient temperature for 13 months [27]. Additionally, at a higher temperature of approximately 40 °C, the TFF-containing formulation was stable for 6 months without using any stabilizer during the process. Throughout storage, the aerodynamics and physicochemical characteristics of the compositions were modified by the moisture levels of the powder. Hydroxypropyl methylcellulose (HPMC) cellulose may impart certain moisture upon storage. The capsules absorb a small amount of moisture in the absence of a desiccant and possibly lead to a decrease in the levels of SSA and MMAD. Water plasticization is the general case in the case of amorphous material and is easily susceptible to environmental moisture. The wetness for a longer period on the surface of the capsule leads to the brittleness of the capsule structure. The moisture level and wettability may affect the particle density and fracture level in capsules [28]. Aerodynamic properties were negatively impacted by changes in particle shape and powder features. The particle characteristics suggest variations in the FPF (fine particle fraction), EF (emission fraction), and MMAD (mass median aerodynamic diameter) [19]. A tightly closed container with a desiccant can variably limit the adsorption of moisture and maintain the aerodynamics of the powder prepared by the TFF technique. The TFF powder was stable at 25 °C for 6 months, while at an incremental temperature of 40 °C, the stability was slightly altered. The aerodynamics of the powder and the size, shape, and density of the powder were measured to quantify the stability level at different temperatures. While filling powdered material into the capsule, the sample was dried for a few mins to remove extra absorbed moisture before filling into the capsule. The encapsulation of protein subunits and conversion into dry vaccine powder was demonstrated by Thakkar et al. [29]. The aluminum hydroxide adsorbs OVA with 2% trehalose in dry powder and was prepared by the TFF technique. The reconstitution rate was very high and was stable at 30 and 40 °C for 1 month. After a longer preservation time at 40 °C, the stability deteriorates, and particle aggregates were observed in the reconstituted solution. At 40 °C, aggregation may be due to an increase in the moisture levels in the vial upon storage for 3 months. The stability of OVA-loaded aluminum hydroxide was not affected between 1 and 3 months, and vaccine powder was reconstituted easily. The in vivo performance of OVA-loaded aluminum hydroxide was very high and did not deviate from the anti-OVA antibody production activity. The glass transition temperature of the prepared OVA vaccine was shifted to 120 °C, which imparts stability for up to 3 months under variable temperature conditions. The increase in *Tg* provides an increase in the physical, chemical, and immunological stability of powdered vaccines prepared by the TFF technique [30]. The stability of biological vaccines can be improved by combining medication and excipients with high *Tg*. The high value of *Tg* may eliminate or lower the mobile glassy state. In some instances, the ratio may vary and not be necessary for maintaining the stability of the vaccine. Storage conditions with or without desiccant and type of desiccant used for storage of TFF powder. The characteristics of TFF were analyzed using NMR, suggesting that the drug possesses a strong glass-forming capacity and did not have any possible interaction with LAC. TAC and LAC were miscible at a domain size of 100 nm, even though DSC discovered two *Tg*s, indicating that the two chemicals were not molecularly dispersed. TAC was physically stable despite phase separation and the absence of interactions, and remaining amorphous after being exposed to 40 °C/75% RH for 28 days in an open bottle and 6 months in a closed container [19].

## 4. Application of TFF Technology in the Development of Nanoformulation

### 4.1. Advanced Technique to Improve the Solubility of API

TFF is a quick-freezing technique useful for increasing the rate of dissolution and solubility characteristics of poorly water-soluble drugs. BCS class II and BCS class IV drugs are generally processed or considered in the development of TFF-based formulations. The effect of instant solvent removal by the sublimation process and promoting instant cooling of the poorly soluble drug may change the physical attributes. Niclosamide has a broad spectrum of antiviral activity and is used for the treatment of COVID-19. The major limitation of niclosamide is poor solubility and thereby less dissolution. Niclosamide is processed using the TFF technique to generate dry powder for use in the management of COVID-19. The prepared inhalation dry powder of niclosamide prepared using TFF provides acceptable aerosol performance. The TFF produces a very large concentration of fine powder with acceptable aerodynamic characteristics. The preclinical investigation conducted on Syrian golden hamsters provides efficient pharmacokinetic data and high efficiency for inhalation applications [31]. In another study, TFF technology was used by Carvalho et al. to develop an amorphous rapamycin formulation. TFF produced an amorphous rapamycin formulation using lactose (as a stabilizing agent). The dry powder formulation has high systemic bioavailability because of its improved solubility. The amorphization process increases the rate of dissolution and FPF in the lungs. The aerodynamics were controlled by controlling the particle size of lactose, which offers better aerosolization of powdered material [32]. Various applications of TFF in improving the solubility of poorly soluble drugs are highlighted in Table 2.

PVP and Soluplus are excellent excipients for enhancing the solubility of poorly soluble drugs. The drying technique can potentially support the dissolution criteria of the selected process. Shamma R. et al. investigated the effect of the drying process on the solubilization behavior of spironolactone in the presence of Soluplus and PVP. The porous structures formed during the TFF process are valuable input from the process aspects. Considering the optimum concentration of PVP and Soluplus can effectively form a porous film using the TFF technique and is suitable for drug delivery to pediatrics [38]. Carbamazepine is an antiepileptic drug with very poor solubility characteristics. The TFF technique suitably converts crystalline carbamazepine into amorphized powder with polymer compositions containing HPMC E3, L100-55, and CA. The system helped accelerate bioavailability by improving the rate of solubilization. Due to the presence of HPMC E3, the formulation shows delayed release characteristics. The TFF technique provides suitability for the delivery of carbamazepine through the oral route by improving its solubility characteristics [35]. TFF with other tandem techniques can improve the multiple characteristics of poorly soluble drugs. Sonication along with TFF can improve the solubility characteristics of oleanolic acid. The PVP K30-coated liposomes were prepared by using the sonication method with an advanced ultradrying technique that provides suitable formulation strategies. Bile salt and sodium deoxycholate act as protective components for burst release from liposomes. The presence of a bilayered structure with the support of a rigid coating provided by PVP K30 provides efficacious utilization for the treatment of hepatitis. The oleanolic acid absorption from GIT increases with the increase in the stability in the acidic environment [39].

**Figure 2:**
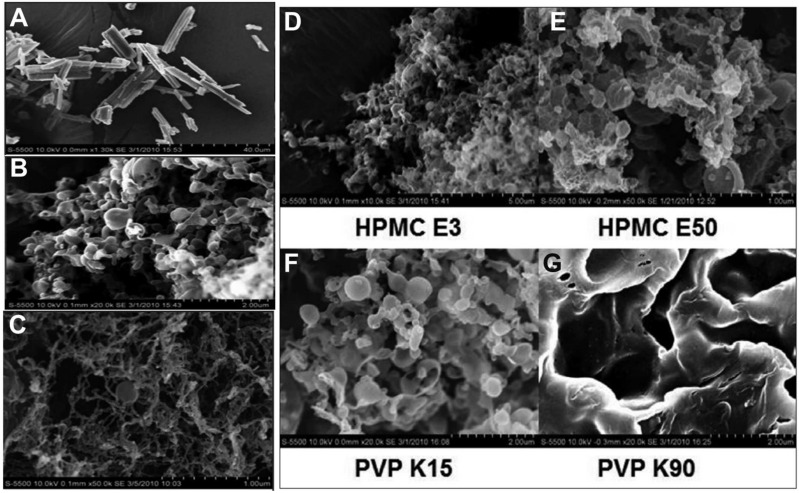
SEM images of (**A**) pure itraconazole (ITZ); (**B**) template emulsion-based ITZ powder; (**C**) cosolvent-processed TFF powder; and template emulsion compositions containing (**D**) HPMC E3; (**E**) HPMC E50; (**F**) PVP K15; and (**G**) PVP K90. Reproduced from Lang et al. [40] with kind permission of copyright holder, Elsevier.

Lang et al. [40] studied TFF with a template emulsion (chloroform and water) and TFF with a cosolvent system (1,4-dioxane, water, lecithin, hydrophilic polymers such as HPMC and PVP) to enhance the wetting and dissolution properties of BCS class II drugs, for example, itraconazole. SEM results indicated that the cosolvent system was highly porous with a matrix-like structure, whereas the template emulsion system was submicron spheres (Figure 2A–C). It was also noticed that the cosolvent system displayed a larger specific surface area ((119.20 m^2^/g) than the template emulsion (39.69 m^2^/g). The contact angle for template emulsion compositions is smaller, thus suggesting better wetting properties. This was proven in dissolution studies, as they showed faster drug release and a greater level of supersaturation. The effects of various hydrophilic components in the cosolvent system are also examined. The composition of the template emulsions changed depending on the hydrophilic polymers chosen. Particles in the HPMC E3-, HPMC E50-, and PVP K15-based formulations had spherical shapes and fell within the submicron size range. The PVP K90-based compound, however, lacked any sphere-like structure. The viscosity of the aqueous solution may be the cause of the formulation’s inadequate emulsification when it is based on PVP K90 (Figure 2D–F). The addition of PVP K-15 and HPMC E-50 showed greater dissolution rates and stable drug supersaturation, respectively. Thus, TFF technology was successfully utilized to enhance the wetting properties of poorly soluble drugs.

### 4.2. Dry Powders for Inhalation

Beinborn and coworkers developed a novel approach, i.e., thin film freezing, a particle engineering technique capable of manufacturing low-density porous aggregate particles, which was used to produce particulate voriconazole formulations with appropriate characteristics for inhalations. These low-density aggregate particles have a large surface area and can be inhaled as a dry powder. The crystallinity and aerodynamic properties of TFF-processed powders were modified by the formulation composition. The inhaled microstructure crystalline voriconazole formulation demonstrated more favorable pharmacokinetics in lung tissue and plasma compared to marketed oral or IV formulations, suggesting that it may be useful in the treatment of infectious lung diseases [37]. In addition, the TFF technique has been used to produce engineered particles with improved powder characteristics that contain voriconazole. The voriconazole formulation by TFF with or without PVP resulted in low-density, brittle matrix particles that could be sheared in situ into respirable particles using a dry powder inhaler for passive inhalation [41]. Moreover, TFF was used to produce aerosolized dry powders of monoclonal antibodies (mAbs) with excellent aerosol performance properties for pulmonary delivery. If tested clinically in the therapy of cancer, the mAb dry powders could have possibly positive benefits [6].

**Figure 3:**
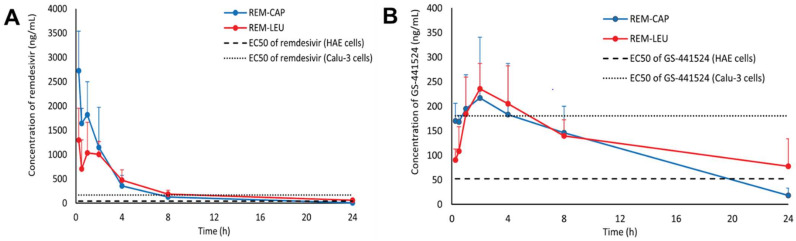
Plasma concentration–time profiles of REM-CAP (remdesivir-Captisol^®^; 80/20 *w*/*w*) and REM-LEU (remdesivir-leucine; 80/20 *w*/*w*) after a single inhalation administration in hamsters; (**A**) remdesivir; (**B**) GS-441524. The dashed line and dotted line represent the EC50 of remdesivir and GS-441524 in human epithelial cells (HAE) and a continuous human lung epithelial cell line (Calu-3), respectively. Reproduced from Sahakijpijarn et al. [46] with kind permission of copyright holder, Elsevier.

TFFD provides a potential approach for preparing dry powders of siRNA-encapsulated solid lipid nanoparticles for inhalation lung delivery [42]. The feasibility of utilizing TFFD to generate aerosolized, porous, and fluffy brittle matrix dry powder of solid lipid nanoparticles for putative pulmonary application was shown in this work. The obtained results demonstrated that thin-film freeze-drying of TNF-α siRNA-encapsulated solid lipid nanoparticles resulted in an aerosolized dry powder with excellent aerosol performance parameters while preserving the physical features of the siRNA-encapsulated SLNs and the function of the siRNA in the SLNs. The TFF approach was used to produce voriconazole nanoaggregates with mannitol as a surface texture modifier. The fine particle fraction and formulation stability were improved in the 3% *w*/*w* mannitol formulation up to the 13th month at 25 °C/60% RH [43]. Furthermore, a group of researchers demonstrated that TFF can be used to manufacture dry powder formulations of protein-based therapeutics, enabling cold chain-free storage and effective pulmonary delivery. TFF can also be used to make dry protein powders for pulmonary administration. Proteins that have been exposed to TFF and sublimated to remove the frozen solvent can maintain their structure and function. Protein dry powders produced via TFF provide excellent thermostability and aerosol characteristics for pulmonary administration [4]. Aboulfotouh and colleagues have studied the suitability of using TFFD to design dry powder of AS01B liposomal adjuvant alone or in combination with AS01B. The particle size distribution of the reconstituted dry powder was identical to that of the liquid adjuvant before drying. When the AS01B liposomal adjuvant was subjected to TFFD with sucrose 4% *w*/*v* as a stabilizer, TFFD seemed to not affect the AS01B/OVA vaccine’s AS01B liposome integrity or immunogenicity. TFFD allowed the manufacturing of dry powder AS01B liposomal adjuvant and AS01B-adjuvanted vaccines using a single stabilizing agent at a low dose [44]. TFFD converted bivalent Norovirus vaccines from liquid suspension to dry powder. Using cryoprotectants such as trehalose or sucrose, the Norovirus vaccine was transformed into a dry powder, and TFFD was given without loss of antigen or particle aggregation while keeping antigen potency within a defined range. According to Xu et al., the dry powder Norovirus vaccine does not require a cold chain during storage and transit [45]. In another pharmacokinetics study, two formulations of remdesivir were prepared, remdesivir-captisol and remdesivir-leucine. Drug and excipients were dissolved in a mixture of acetonitrile/water and subsequently dried to deliver remdesivir to the lung as a dry powder. Remdesivir was converted into GS-441524 in the lung as well as in the plasma of hamsters. Both levels in plasma are sufficient to produce antiviral action. Remdesivir-leucine (80/20 *w*/*w*) had a higher C_max_ with a relatively short T_max_ and lower AUC of GS-441524, implying that reduced drug exposure is needed for a high effective concentration against SAR-CoV-2, although the Remdesivir-Captisol^®^ (80/20 *w*/*w*) (Ligand Pharmaceuticals, San Diego, CA, USA) formulation demonstrated quicker and higher uptake of remdesivir and GS-4412524 in the lung (Figure 3). For the therapy of COVID-19, remdesivir dry powder for inhalation would be a potential alternate dosage form [46].

In another study, remdesivir dry powder was prepared with the help of TFF techniques to maximize the lung delivery of remdesivir for SARS-CoV-2. The obtained brittle nanostructured aggregates are sheared into respirable low-density microparticles after aerosolization from a passive dry powder inhaler. Remdesivir along with optimal excipients show desirable aerosol performance. The TFF process is suitable to produce an amorphous form of remdesivir, helping drug dissolution in simulated lung fluid. During the in vivo study, remdesivir leucine was poorly absorbed and had a lower plasma concentration than remdesivir captisol. Remdesivir is a prodrug and is dependent on GS-441524 for absorption. If the concentration of GS-441524 is optimum, the formulation can be well absorbed [14]. In another study, the TFF process was used to produce highly stable submicron lactate dehydrogenase (LDH) and lysozyme particles followed by lyophilization. Protein particles formed through TFF have an average diameter of 300 nm and show 100% enzyme activity after reconstitution for LDH. The intermediate cooling rate regime for TFF offers a promising route to form stable submicron protein particles of attention in parenteral as well as pulmonary drug delivery [11]. Similarly, paclitaxel-containing freeze-dried liposomes with gelatin support were prepared by thin-film dispersion as well as the freeze-drying technique. Various characteristics of GLs, including entrapment efficiency, particle size, and gelation temperature, were broadly evaluated. The stability of liposomes was improved by interior gelatin support [47]. Table 3 represents the use of TFF technology for preparation of DPI formulations using different types of stabilizers or polymers.

## 5. Recent Advancements of Thin Film Freezing in Novel Drug Delivery Systems

TFF technology has been extensively studied for novel drug delivery systems (NDDSs) suitable for converting liquid NDDS formulations into dry powders with the loading of many therapeutic agents, including remdesivir, tacrolimus, niclosamide, voriconazole, siRNA, and vaccines. The development of TFF-processed NDDS dry powders has the potential to overcome issues associated with stability and carrier as a critical component of the formulation [53].

### 5.1. TFF Processed DPI for SARS-CoV-2 (COVID-19)

SARS-CoV-2 (COVID-19) has created havoc among people from all over the world, and many drugs were introduced between early and mid-2021 to treat the SARS-CoV-2 virus. Since COVID-19 is associated with the respiratory system, inhaled formulations can be found to be more efficient and less toxic. In clinical trials, most antiviral drugs against SARS-CoV-2 showed uneven outcomes or limitations [53]. For instance, remdesivir metabolizes quickly and shows many adverse effects, such as ECG variations and liver and renal toxicity. Favipiravir also showed some adverse effects and drug interactions, and it was also observed that the concentration of the drug was reduced after prolonged administration. Hydroxychloroquine was absorbed variably, showing QT prolongation in the patients. To resolve these issues, drugs that can be inhaled were administered. Inhalation of azithromycin-loaded polycaprolactone microparticles displayed promising properties that might assist in the treatment of lung infections on a local level. The outbreak of the COVID-19 virus has increased the amount of research and repurposing of drugs so that an effective solution or remedy can be found. One such solution was to produce dry powder for inhalation of remdesivir via the thin film freezing method. In comparison to microparticles and nanoaggregates, TFF-processed DPI exhibits a greater drug absorption efficiency and dosage uniformity in the lungs. Additionally, this process can increase the solubility profile of poorly aqueous soluble drugs and, as a result, enhance bioavailability. TFF also shows an ultrarapid freezing rate so that particle size can be controlled [54].

Remdesivir DPI was developed with a combination of excipients, including Captisol^®^, mannitol, lactose, and leucine, using the TFF method and exhibited acceptable aerosol performance (up to 93.0% FPF < 5 µm and 0.82 µm MMAD) with one month of stability at 25 °C/60% RH [14]. Remdesivir remained in an amorphous form and displayed 20 times higher solubility in simulated lung fluid with TFF-processed Remdesivir–Captisol^®^/lactose DPI in comparison to Remdesivir–leucine/mannitol DPI due to its crystalline form. Moreover, in vivo pharmacokinetic investigation revealed improved absorption of the Remdesivir-Captisol^®^ DPI formulation from the lungs to systemic circulation. Considering this, TFF-processed DPI is expected to extend to other drugs and pave the way for developing formulations for COVID-19 treatment.

In a recent study, Robert O. Williams III employed thin-film freezing (TFF) to develop niclosamide dry powder inhalation (DPI), and further pharmacokinetic and toxicological studies were performed in rats and hamsters [31]. TFF-based inhalation powder technology has been of enormous interest in lung-specific targeting. In this study, the DPI form of niclosamide (an anthelmintic drug) was developed since studies elaborated upon its potential as a broad-spectrum antiviral, including coronaviruses (SARS-CoV-2). These niclosamide-based powders displayed an acceptable aerosol performance with a fine particle fraction (FPF) (86.0 ± 2.7%) and MMAD of 1.11 µm. The aerosol performance of this niclosamide inhalation powder was determined and found to be higher (FPF or delivered dose is 86.0 ± 2.7%) than that of niclosamide-based powder inhaler technologies, including spray-drying techniques. Later, the histopathological study confirmed the safety of these powders after three days, multidose tolerability, and exposure studies in rats. After a single administration of these DPIs to Syrian golden hamsters, the niclosamide concentration achieved above IC90 levels for at least 24 h. Thus, the poor oral bioavailable drugs can be surpassed by their direct administration into the lungs via TFF-based dry powder inhalation formulation.

### 5.2. TFF-Processed DPI for Tacrolimus Delivery

Another study by the same group formulated tacrolimus (TAC) dry powder inhalation (DPI) using TFF technology to reduce the amount of lactose (LAC) for the development of powder [19]. In this study, TFF-TAC-LAC displayed high drug loading (95%) and acceptable aerosol performance without affecting dissolution and stability. These properties have been achieved because of the unique properties of TFF powder and the glass-forming ability (class III glass forming API former) of TAC. The solid content and processing temperature did not affect the aerosol performance of tacrolimus. After 6 months of storage at 40 °C/75% RH and 25 °C/60% RH, the amorphous state was retained, and aerosol performance did not change significantly. The authors extended this study to understand the safety and tolerability of TAC-LAC as a dry powder and suspension form in healthy human subjects [20]. This study proved the safety and tolerability of healthy human volunteers after administration (3 mg/dose) of TAC-LAC formulations.

In another investigation, both amorphous and crystalline forms of tacrolimus-loaded DPI were developed by TFF and micronization methods, respectively. The TFF-processed DPI formulation exhibited 83.3% FPF and 2.26 μm MMAD compared with the 78.5% FPP and 2.08 μm micronized DPI formulations. This resulted in higher deposition and extensive drug retention in the tissues of the lungs with TFF-based DPI formulations. As reported by Watts et al., since TFF-processed powders with mannitol remained uninfluenced by high humidity, they were chosen for study. TFF particles are preferred over micronized TAC-MAN since they undergo rapid dissolution, have a high permeability through the airway membrane, and can escape macrophage phagocytosis, hence increasing pulmonary bioavailability and reducing adverse side effects. As reported by Watts et al., since TFF-processed powders with mannitol remained uninfluenced by high humidity, they were chosen for the study. TFF particles are preferred over micronized TAC-MAN since they undergo rapid dissolution, have a high permeability through the airway membrane, and can escape macrophage phagocytosis, hence increasing pulmonary bioavailability and reducing adverse side effects [34].

### 5.3. TFF-Processed Bacteriophage Dry Powders

Phage therapy is widely preferred over antibiotic therapy because of its safety, efficacy, improved pharmacokinetic profile, and increasing prevalence of multidrug resistance. Solid (dry) formulations are always better than liquid formulations because of many factors (mostly stability). The researchers used the T7 phage model to develop dry powder bacteriophage via TFF without significantly losing its viability. This method overcomes all the processing challenges and is hence desirable for preserving heat and mechanically sensitive therapeutic moieties. The excipients used for solid phage formulations are sugars (lactose, sucrose, trehalose) in combination with amino acids (leucine). After TFFD, the compositions of sucrose and trehalose were amorphous. The right ratios of sugar and leucine can give stable and desirable phage formulations. It was observed that by adding buffers, the particle size was significantly reduced, which indicates that the presence of a buffer system in the solutions significantly improves the survival of phages during the freezing-sublimation process [48]. Thus, phage powders created by TFFD have much promise for use in respiratory administration.

### 5.4. TFF-Processed Dry Powder for Pulmonary Fungal Infection

**Figure 4:**
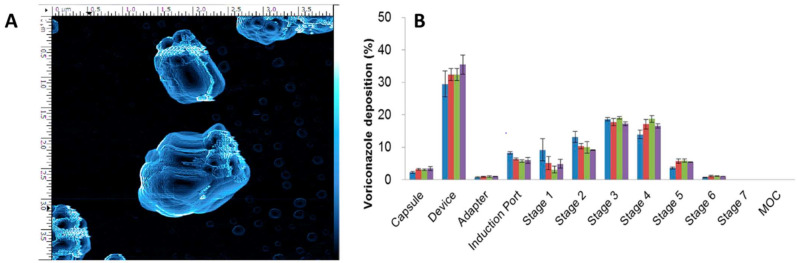
(**A**) AFM topography image of aerosolized TFF-VCZ-MAN 95:5 by DP4 insufflator; (**B**) Aerodynamic particle size distribution profile of TFFVCZ-MAN 95:5 by time sheared: (blue) at 0 min; (red) at 15 min; (green) at 30 min; (purple) at 60 min (*n* = 3; mean ± SD). Reproduced from Moon et al. [27] with kind permission of copyright holder, American Chemical Society.

Moon et al. [27] discussed nanoaggregates of voriconazole (VCZ) as the first-line treatment of invasive pulmonary aspergillosis (IPA), which has a high mortality rate. This investigation suggests and further proves that adding a smaller number of excipients in nanoaggregates for aerosol formulation enhances the potency. TFF was used to formulate nanoaggregates (Figure 4A) as an ultrarapid freezing technology with a freezing rate of up to 10,000 K/s. Mannitol (MAN) is used as an excipient (from the GRAS excipient list), which provides porosity to the matrix of nanoaggregates; it also modifies the surface texture of nanoaggregates and hence provides better dissolution rates and faster wetting properties. The developed voriconazole DPI showed high aerosol performance (Figure 4B) with 73.6 ± 3.2% FPF and an MMAD of 3.03 ± 0.17 μm with a low amount of mannitol (3% *w*/*w*).

The authors have put forward a scale-up strategy for voriconazole nanoaggregates. TFF overcomes the drawbacks of lyophilization, as it is quick cooling; thus, homogenous nucleation occurs with a small size of solvent droplets regardless of the scale. Process design parameters are established with formulation TFF-VCZ: MAN (95:5) over different temperatures and flow rates. Large- and small-scale manufacturing showed FPFs (% metered dose) and MMADs of 35.6% vs. 37% and 3.69 µm vs. 3.52 µm, respectively. For a lower process temperature (−150 °C), there was a significant increase in FPF because a lower temperature leads to faster cooling, quick nucleation, and thus the generation of finer nanoparticles (200 nm). Other important parameters that affect the process are viscosity and cryo-phase separation of the solvent system. There were no substantial changes in the FPF with a variable loading dose. A successful 450-fold scale-up was achieved with high potency surface texture modification [52].

### 5.5. TFF Technology for Solubility Enhancement

**Figure 5:**
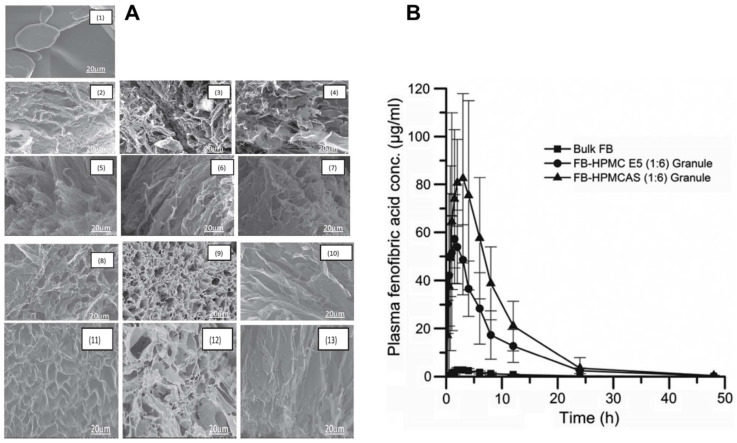
(**A**) SEM micrographs of bulk FB and FB solid dispersions prepared by the TFF process. (1) Bulk FB; (2) FB-Soluplus (1:4); (3) FB-Soluplus (1:6); (4) FB-Soluplus (1:8); (5) FBHPMC E5 (1:4); (6) FB-HPMC E5 = 1:6; (7) FB-HPMC E5 = 1:8; (8) FB-HPMCAS (1:4); (9) FB-HPMCAS (1:6); (10) FB-HPMCAS (1:8); (11) FB-HP55 (1:4); (12) FB-HP55 (1:6); and (13) FB- HP55 (1:8); and (**B**) Average plasma-drug concentration of fenofibric acid following single-dose oral administration of different formulations to Wistar rats (data presented are mean ± SD, *n* = 6, dose is 27 mg/kg). Reproduced from Zhang et al. [3] with kind permission of copyright holder, Elsevier.

Zhang et al. [3] developed a TFF-processed fenofibrate (FB) solid dispersion by using 1,4-dioxane as a solvent and with different ratios of excipients (Soluplus^®^, HPMC). The polymer having a stabilizing effect for the supersaturated solutions can deliver enhanced FB dissolution with either physical or chemical interactions. Herein, to examine the surface characteristics of the samples, SEM was used. Substantial, dense crystals at the micron size may be seen in the image of bulk FB. On the other hand, the FB solid dispersion geometry was found to be extremely porous (Figure 5A). Together with neighboring aggregates, the porous microparticulate aggregates formed a sponge-like structure. Due to the quick-freezing method used to create the solid dispersions and the polymers’ capacity to prevent crystal formation, no FB crystals were seen because of the uniform blending of FB and polymers. The 1:6 ratio of FB: HPMC was found to be the best for dissolution testing. The HPMC-based formulation had greater supersaturation than the Soluplus^®^ formulation, which may be due to its ionized form above pH 5 that stabilizes the aggregates. The polymer aggregates showed higher drug plasma concentrations and higher supersaturation in vitro than pure FB (Figure 5B). Thus, the study provides TFF-processed FB formulations with enhanced performance in vitro and in vivo.

### 5.6. CRISPR–Cas9 Nanocomplexes in DPI

For the treatment of genetic disease and cystic fibrosis, researchers developed PEGylated chitosan/CRISPR–Cas9 nanocomplex-based dry powder inhalers using the TFF technique with different cryoprotective agents (mannitol, sucrose, and trehalose) and dispersion enhancers (leucine) [49]. In comparison with other cryoprotectants, DPI formulations containing 3% mannitol with leucine (DPI-ML) and without leucine (DPI-M) displayed acceptable aerodynamic performance, with MMADs of 4.8 and 4.6 μm, respectively. These properties are essential for particle deposition in the lungs. The DPI with 0.5 to 3% cryoprotectants showed the highest transfection efficiency compared to low and high cryoprotectant concentrations. Similarly, formulations without any cryoprotectant observed low transfection efficiency. This study concluded that TFF-processed CRISPR–Cas9 DPI could be a promising way to deliver gene-editing therapeutics using pulmonary delivery that enables noninvasive and direct deposition of drugs in the lungs.

### 5.7. TFF Processed siRNA Encapsulated Solid Lipid Nanoparticles

**Figure 6:**
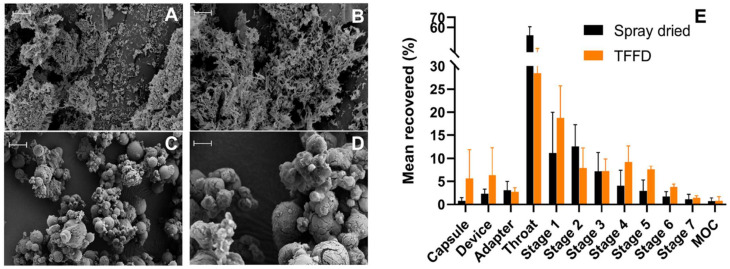
Representative SEM images of dry powders of SLNs prepared by thin-film freeze-drying (**A**,**B**) or spray drying (**C**,**D**). In (**A**,**C**), the scale bar indicates 10 µm, and in (**B**,**D**), the scale bar indicates 2 µm; (**B**) deposition patterns of dry powders of SLNs prepared by spray drying or thin film freeze-drying in NGI (MOC, micro-orifice collector). Data are the mean ± S.D. (*n* = 3). Reproduced from Wang et al. [42] with kind permission of copyright holder, Elsevier.

In another study, siRNA-encapsulated solid lipid nanoparticles (SLNs) were developed for pulmonary delivery using the TFF method and compared with spray-drying (SD) and freeze-drying (FD) techniques [42]. In this study, mannitol was used as a cryoprotective agent because of its better aerosol performance property and osmotic property. It was found that TFF-processed DPI resulted in a 20 times greater surface area with less moisture content than dry powder prepared by SD. The dry powder of SLNs produced by TFFD had a porous appearance and was fluffy and brittle (Figure 6A,B), whereas the dry powder produced by spray drying had a microstructure resembling beads (Figure 6C,D). In addition, dry powder by TFF showed better aerosol performance (Figure 6E) and preserved the function of siRNA in SLNs, which indicates that TFF techniques could be a promising tool to deliver lipid nanoparticle dry powder for pulmonary delivery.

### 5.8. TFF Processed Dry Vaccine Powders

**Figure 7:**
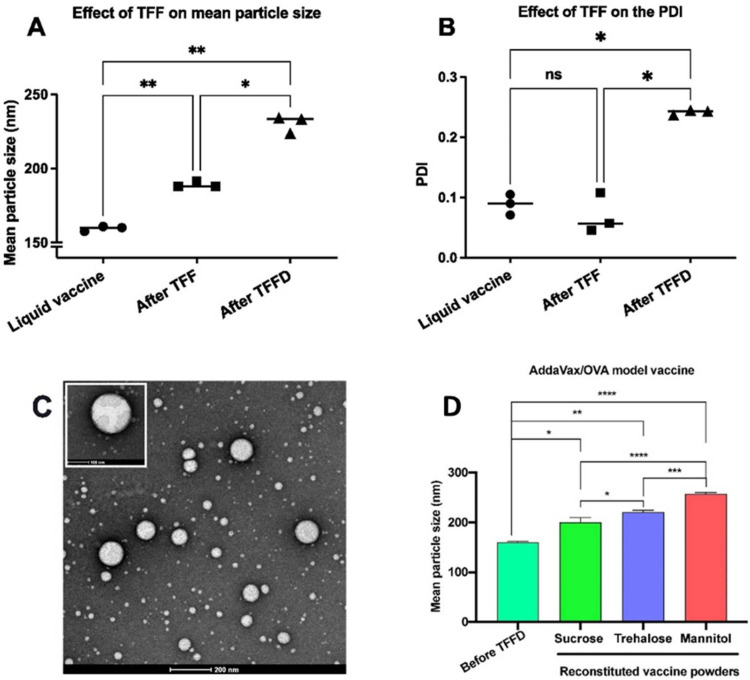
Effect of the freezing and drying steps of TFFD on (**A**) the mean particle size; and (**B**) PDI of AddaVax/OVA vaccine; (**C**) representative TEM image of AddaVax/OVA vaccine reconstituted from a thin-film freeze-dried powder (the scale bar is 200 nm). * *p* < 0.05, ** *p* < 0.01, ns: non-significant (*p* > 0.05); (**D**) effect of stabilizing agent and its concentration on the mean particle size of AddaVax/OVA model vaccine. The efficiency of sucrose, trehalose, and mannitol * *p* < 0.05, ** *p* < 0.01, *** *p* < 0.001, **** *p* < 0.0001, *ns*: non-significant (*p* > 0.05). Reprinted with permission from [55].

In a recent study, TFFD was used for converting liquid AddaVax™ (MF59 nanoemulsion adjuvant) to dry powders used in H1N1 influenza vaccines. TFF-processed AddaVax™ dry powder with trehalose showed an acceptable droplet size distribution without any changes in their function, while freeze-dried powder displayed large particles. Therefore, TFF presents a novel and effective process for converting liquid vaccines into dry powder vaccine formulations (see Figure 7) [55]. Furthermore, the processing steps are crucial in deciding the PDI and particle size of the particles.

## 6. Commercial Prospects and Future Scope of TFF

Thin film freezing (TFF) produces a stable form of powder drugs and biologicals (vaccine, enzymes, proteins, and antibodies) with improved solubility and has potential for oral delivery and with less stringent storage conditions. Biologics and vaccines in solution are directly converted into a powder, mostly used for lung diseases such as fungal infection, bacterial infection, lung dysfunction, and pneumonia. In the future, TFF will have the prosperity to explore other applications of biologicals to treat diseases of the airways and gastrointestinal tract. Thin film freezing particulate engineering technology designed for the formulation of dry powders to improve the solubility of poorly water-soluble drugs for improving bioavailability and absorption technology is explored for targeted inhalation delivery for pulmonary diseases in deep lungs and airways. Low bulk density, amorphous nature, high surface area, and porosity have the potential for contacting the target site of the lungs for the intended effect. The incorporation of aerodynamic properties into the powder dosage form is always challenging in drug delivery methods. TFF showed potential and is used for stabilizing vaccines, antivirals, small molecules, biologics, and antibodies. For clinical translation, TFF technology is in the final stage. This technology is used for designing particulate powder for respiratory diseases, including pulmonary fibrosis and COVID-19. During the pandemic, emerging techniques such as TFF are useful for improving the stability of biological products along with improving therapeutic efficiency. The plasmid DNA delivery application of TFF has shown promising results. In the literature, powders of dry powder of *E. coli* and *L. acidophilus* have been successfully developed with TFF with minimum bacterial viability loss, and *L. acidophilus* dry powder is suitable for intranasal delivery revolutionizing drug delivery and the biopharmaceutical field. Validation of effectiveness is a critical step for TFF technology transfer. TFF powdered technology is easily scalable, simple, affordable, has thermal stability and is stable at shear stress and denaturation. Encapsulated TFF powders in dry powder inhalers showed 80% drug deposition into the lungs with minimum loss and degradation. In the future, the power obtained by TFF can be used by other routes of administration, such as intranasal, intraocular, and transdermal administration. In 2022, voriconazole and tacrolimus were in phase II clinical trials for invasive pulmonary aspergillosis and lung transplant rejection, respectively. The U.S. FDA approved the orphan drug TAC-LAC for lung allograft rejection prophylaxis. Despite the clinical success of TFF, physical and chemical instability and moisture sensitivity are the major challenges that need to be addressed for future developments to minimize excipients in formulation, viability, and cost-effectiveness for patients.

## 7. Conclusions and Future Perspectives

Particle engineering technology provides alternative methods of continuous manufacturing protocols for pharmaceutical industries. Particle engineering, such as thin film freezing techniques, is not only used in pharmaceuticals but is equally important for the long-term preservation of biological material, food ingredients, nutraceuticals, etc. Fundamental aspects of TFF techniques generate fine, freely flowable particles by applying the solution to cryogenically cooled surfaces. The physicochemical characteristics of freeze-dried particles by TFF produce a stable solution useful for the direct delivery of biomolecules, vaccines, antibodies, proteins, amino acids, labile drugs, etc. Applications of TFF techniques open new avenues in the delivery of solid dry powder materials, which provides ease for transportation and long-term storage and facilitates the enhancement of bioavailability. However, the traditional method for product development necessitates considerable time consumption and difficult trial-and-error experimentation. Machine learning has acquired significant popularity in pharmaceutical research as a cutting-edge approach and has been extensively used in a variety of contexts. These machine learning models could facilitate the DPI product development process and have the potential to significantly minimize the burden associated with product development. In the future, the techniques can be coupled with a continuous manufacturing process for the synthesis of nanoparticles, liposomes, dry powder inhalers, etc. The continuous process of manufacturing is demanding and provides automation with a reduction in errors. Continuous automated manufacturing facilities deliver high-end accurate output and are an important technique for pharmaceutical industries. The process and product parameters can be controlled by the single-handed operator and provide a uniform product. The review emphasizes the fundamental and stability aspects of thin film freezing techniques for the delivery devices of poorly soluble drugs using novel carriers.

## Figures and Tables

**Table 1:** Summary of enabling technologies to produce DPI [1,13].

	TFF	Spray Drying	Spray Freeze Drying	Nano Milling
Potential to prevent molecular damage				
**Thermal Degradation**	Yes	No	Yes	No
**Shear Stress**	Yes	No	No	No
**Air/water denaturation**	Yes	No	No	Yes
**Technology Differentiation**	Nanoaggregate particles (higher absorption) Higher yield Uniform particle size Good for labile molecules	Microparticles Lower yield Harsh processing conditions	Microparticles Lower yield Wider particle size distribution (Improper for DPI) Not suitable for viscous solution	Microparticles Crystallinities exist (insoluble in lungs) Harsh processing conditions
**Limitations of technology**	Lower surface area/volume ratio Process stabilization	Potential protein denaturation	High air/water interface Tedious scale up and high cost	Inability to induce complete amorphization

**Table 2:** TFF Technology for low solubility API.

Drug/API	Excipients	Formulation	Application of TFF Technology	Ref.
Fenofibrate	HPMC, CMC-Na, Methocel, Soluplus	Dry powders/Amorphous solid dispersions	The TFF approach demonstrated improved dissolving rates, which in turn improved the drug’s bioavailability since it was not very water soluble.	[3]
Niclosamide	Mannitol, and leucine	Inhalation dry powder	Resolves the bioavailability restriction of niclosamide. Satisfactory aerosol efficiency was displayed.	[31]
Micronized danazol/bulk API	Polyvinylpyrrolidone	Nanostructured amorphous API	The production of weakly water-soluble APIs into high surface area nanostructured particles with quick dissolving rates using URF technology is a practical and reliable procedure that will probably increase the APIs’ accessibility in vivo.	[2]
Rapamycin	Lactose	Crystalline dry powder formulations	Comparing the physical mixture composition to the rapamycin formulation, the latter showed improved in vitro aerodynamic characteristics, exhibited quicker dissolving rates and improved solubility, both of which resulted in increased in vivo systemic bioavailability.	[32]
Itraconazole	Hypromellose acetate succinate LF	amorphous solid dispersions	Improved dissolving capabilities in the ITZ amorphous solid dispersion formulation could further boost the drug’s oral bioavailability.	[33]
Tacrolimus	Lactose, Mannitol, Trehalose	dry powder for inhalation/nanostructured aggregates	DPI formulations increase solubility and offer good aerosolization.	[19]
OVA-Alhydrogel (Aluminum Salt-adjuvanted Vaccine)	Trehalose,	vaccine dry powder	Improved solubility and stability	[29]
Tacrolimus	Mannitol	DPI	Substantial increase in pulmonary bioavailability.	[34]
Tacrolimus	Lactose	inhaled formulations/colloidal dispersions after reconstitution and as a dry powder, DPI	For patients who have received lung transplants, pulmonary administration of TFF TAC-LAC could be a safe and effective treatment option.	[20]
Carbamazepine	HPMC E3, L100-55, cellulose acetate (CA)	modified-release amorphous solid dispersions (mr-ASD)	In the CBZ-mr-ASD compositions, the absorption of CBZ and its primary active metabolite, CBZ-E, considerably increased.	[35]
Itraconazole	1,4-dioxane	-	AFD’s reduction in chargeability is greatly desired since it enables better powder management.	[36]
Voriconazole	Polyvinylpyrrolidone	Dry powder insufflation	Improved solubility and dissolution rate.	[37]

**Table 3:** Dry powder for inhalation prepared by TFF technology.

Drug/API	Excipient	Formulation	Application of TFF Technology	Ref.
Voriconazole	Mannitol	Nanoaggregates of crystalline voriconazole powder/DPI	Drug and therapy alternatives with enhanced aerosol performance and excellent aerosolization efficacy for pulmonary aspergillosis invasion.	[27]
Bacteriophage-T7 phage	sucrose and leucine	Dry powder inhalers	Stabilize phage	[48]
PEGylated chitosan/CRISPR–Cas9 polymer nanocomplexes	mannitol, sucrose, trehalose, leucine, chitosan, etc.	DPI	Two preparations with 3% mannitol, either containing or without leucine, were discovered to be appropriate for inhalation and to have the requisite aerodynamic properties	[49]
Bovine serum albumin	HFA 227, 2H, 3H-Perfluoropentane	Nanorods	When pMDIs are used to actuate nanorods, which are stable against settling, the nanorods can be generated to create high fine particle percentages and ideal aerodynamic diameters.	[50]
tetanus toxoid vaccine	aluminum hydroxide, trehalose	Dry powders	By adding aluminum salt adjuvant to dry vaccine powder, the TFFD approach can be used to create new vaccines or reformulate old ones.	[51]
Voriconazole	lactose monohydrate, PVP	DPI, Nanostructured, Amorphous Solid Dispersions	When inhaled passively through DPI, the low density, brittle matrix components in the TFF formulation could breakdown in place to form inhalable particles.	[41]
Monoclonal antibodies anti-PD-1 mAbs	Mannitol, lactose, Trehalose, Polyvinylpyrrolidone	Aerosolizable dry powders	Increased stability in storage, enhanced aerosol efficiency	[6]
Aerosolizable siRNA/TNF-α siRNA	Lecithin, Mannitol	Dry powder solid lipid nanoparticles	Excellent aerosol performance characteristics and maintain the SLNs’ physical characteristics after siRNA encapsulation.	[42]
Voriconazole	Mannitol	Nanoaggregates for dry powder inhalation	TFF was used to create crystalline voriconazole nanoaggregates at various inhalation flow rates and medication loadings.	[52]
voriconazole	Mannitol	voriconazole nanoaggregates	Up to the 13th month at 25 °C/60% RH, the FPF and formulation stability of the 3% *w*/*w* mannitol composition increased.	[43]
Protein	-NA-	protein-based therapeutics	To enable cold chain-free storage and efficient pulmonary distribution, protein dry powders manufactured by TFF have high thermostability and aerosol properties.	[4]
AS01B	-NA-	Dry powder	TFFD permitted the use of a single stabilizing agent at a low dosage to produce both dry powder AS01B liposomal adjuvant and AS01B-adjuvanted vaccines.	[44]
Bivalent Norovirus vaccines	trehalose or sucrose	Dry powder	A cold chain is not necessary for the norovirus vaccine while it is being stored and transported.	[45]
Rapamycin	Lactose	Respirable rapamycin powder	TFF products dramatically increased uptake and presystemic elimination absorption.	[32]
Remdesivir	Leucine, Captisol	dry powder insufflation/inhalation,	Drug concentration in plasma is sufficient to provide antiviral action. Expand the COVID-19 therapy	[46]
Remdesivir	Captisol, mannitol, lactose, and leucine	dry powder for inhalation	Antiviral medication can lessen linked morbidity and mortality by enhancing physical stability.	[14]
ovalbumin (OVA)-Alhydrogel	Trehalose, aluminum (oxy) hydroxide	dry powder vaccine/Intranasal vaccination by dry powder, nasal dry powder delivery device	Improved stability of storage. The flow characteristics of dry vaccine powder are excellent. The dry powder vaccines were evenly distributed.	[30]
LDH or lysozyme/lactate dehydrogenase (LDH)/Protein Particle	Trehalose	Dried Powders	A promising approach to create stable submicron protein particles of interest in respiratory and parenteral administration uses is the intermediate cooling rate regime for TFF.	[11]
Tacrolimus anhydrous	α-lactose, mannitol, and raffinose	Dry Powder inhalation	TFF approaches will be helpful for producing aerosolized brittle matrices and a viable platform for thermolabile, highly powerful, and poorly water-soluble drug delivery to the lungs.	[28]
